# Efficacy and safety of moxidectin and albendazole compared to ivermectin and albendazole co-administration in adolescents infected with *Trichuris trichiura*: a randomized controlled trial protocol

**DOI:** 10.12688/gatesopenres.13299.2

**Published:** 2021-09-27

**Authors:** Sophie Welsche, Emmanuel C. Mrimi, Ladina Keller, Eveline Hürlimann, Daniela Hofmann, Jan Hattendorf, Said M. Ali, Jennifer Keiser

**Affiliations:** 1 Swiss Tropical and Public Health Institute, Basel, Switzerland; 2 University of Basel, Basel, Switzerland; 3 Public Health Laboratory Ivo de Carneri, Chake Chake, Pemba, Tanzania

**Keywords:** Trichuris trichiura, Drug efficacy, Drug safety, Tanzania, Ivermectin, Moxidectin, Albendazole, Soil-transmitted helminthiasis

## Abstract

**Background: **Infections with soil-transmitted helminths (STHs) predominantly affect impoverished populations in tropical environments. The periodic administration of single dose benzimidazoles (i.e., albendazole, mebendazole) to at-risk individuals in endemic regions is at the center of STH control strategies. Given the low efficacy of these drugs against trichuriasis, investigation of drug combinations including moxidectin and ivermectin has recently been initiated, yet the identification of the best treatment option requires more research. We present the protocol for a trial investigating the efficacy and safety of co-administered moxidectin and albendazole compared to co-administered ivermectin and albendazole against *Trichuris trichiura*.

**Methods:** We will conduct a randomized controlled trial enrolling 540 *T. trichiura*-infected adolescents aged 12-19 years on Pemba Island (Tanzania). The trial will be open-label with blinded outcome assessors. The primary objective is to demonstrate non-inferiority of orally co-administered single-dose moxidectin (8 mg)/albendazole (400 mg) compared to orally co-administered single-dose ivermectin (200 µg/kg)/albendazole (400 mg) in terms of egg reduction rates (ERRs) against *T. trichiura* infections assessed by Kato-Katz at 14-21 days post-treatment. Secondary objectives include the assessment of the drug combinations’ superiority compared to their respective monotherapies, of the cure rates (CRs) against *T. trichiura*, and the safety and tolerability of all treatments, as well as CRs and ERRs against concomitant STH infections ( *Ascaris lumbricoides* and hookworm). Potential effects of the treatment regimens on follow-up prevalences of STH at 5-6 weeks and 3 months post-treatment and pharmacokinetic/  pharmacodynamic parameters will also be assessed.

**Conclusions:** Results from this trial will help to inform decision- and policymakers on which anthelminthic combination therapy might improve existing deworming programs and provide a valuable adjunct tool for interrupting STH transmission.

**Clinicaltrials.govregistration:** NCT04700423 (07/01/2021)

## Introduction

Albendazole and mebendazole are comprehensively used in preventive chemotherapy campaigns to mitigate soil-transmitted helminth (STH) infections. The two benzimidazoles show high cure rates (CRs) against infections with *Ascaris lumbricoides* (both Albendazole and Mebendazole CR 96%) and moderate results against hookworm infections (Albendazole CR 80%, Mebendazole CR 33%). Against *Trichuris trichiura* infections however, neither of them are efficacious (Albendazole CR 31%, Mebendazole CR 42%) and thus fall short in achieving the World Health Organization (WHO) goals of morbidity reduction ^1, 2^.

Therapies combining two or more drugs are common in several other treatment areas to protect against drug-resistance as well as increasing and broadening the efficacy in comparison to single treatments ^3^. The combination of ivermectin/albendazole was added to the WHO Essential List of Medicines for the treatment of STH infections in 2017 ^4^. At this stage, the moxidectin/albendazole combination was classified as second tier priority because moxidectin had not yet been approved ^5^.

While for ivermectin/albendazole evidence of superiority compared to single standard treatments in different settings and over varying time points is mounting ^6, 7^, only few studies have examined the co-administration of moxidectin and albendazole in STH infections ^8^ and to date, no head to head comparison between moxidectin/albendazole versus ivermectin/albendazole has been conducted.

Moxidectin recently got approval by the US Food and Drug Administration (FDA) for the treatment of onchocerciasis at an oral single-dose of 8 mg. Clinical trials carried out by our research group have shown that the combination of moxidectin/albendazole might reveal high potential in the treatment of STH infections ^8, 9^. Moreover, the FDA-approved 8 mg dose, both in monotherapy and in combination with albendazole, was found to perform equally well as higher doses of moxidectin against STH species, as shown by our recent dose-finding study against *T. trichiura* infections ^9^. It remains to be explored whether the longer half-life of moxidectin (T _1/2_: 491-832 hours) ^10, 11^ compared to ivermectin (T _1/2_: 16-32 hours; increasing with age) ^12^ might prove beneficial to the treatment of STH infections and long-term outcomes ^13^. The pharmacokinetic/-dynamic (PK/PD) characterization of a drug is essential for the understanding of the human body’s response to a drug and *vice versa*. Physiological characteristics like mal- or undernutrition or infection with intestinal worms such as *T. trichiura* potentially affect the PK of a drug ^14, 15^. For moxidectin, PK properties have been assessed only in a limited number of studies and not yet in *T. trichiura*-infected participants ^16, 17^.

In this paper, we present the protocol for a Phase 3 randomized controlled trial on the efficacy and safety of moxidectin/albendazole combination therapy compared to co-administered ivermectin/albendazole against *T. trichiura* and concomitant STH infections in participants aged 12–19 years. Secondary objectives include the assessment of the drug combinations’ efficacies against *T. trichiura* infections compared to monotherapies, as well as the investigation of potential extended effects through a prolonged efficacy assessment scheme (i.e., follow-up at 14–21 days, 5–6 weeks and 3 months post-treatment).

### Research objectives

We designed a randomized controlled trial to show non-inferiority of co-administered moxidectin/albendazole compared to co-administered ivermectin/albendazole in participants aged 12–19 years on Pemba Island, Tanzania. From evidence of previous studies conducted by our research group, we expect similar efficacies from the moxidectin/albendazole combination compared to ivermectin/albendazole ^8, 9^. Nevertheless, moxidectin might be advantageous in terms of the drug’s longer half-life and as an alternative in areas with possible emerging ivermectin resistance ^16, 18^. As recommended for new combination therapies, we simultaneously assess superiority of the drug combinations compared to monotherapies.

The primary objective is to demonstrate non-inferiority of co-administered moxidectin (8 mg)/albendazole (400 mg) compared to combined ivermectin (200 µg/kg)/albendazole (400 mg) in terms of egg reduction rates (ERRs) against *T. trichiura* infections assessed by Kato-Katz at 14–21 days post-treatment in adolescents aged 12–19 years with a non-inferiority margin of 2 percentage-points and 90 power at the 95% confidence interval.

The secondary objectives of the trial are:

a) to demonstrate superiority against the respective monotherapies in terms of CRs against *T. trichiura* infections assessed by Kato-Katz 14-21 days post-treatment, as this is required for efficacy assessments of combination therapies. Therefore, the trial has five different treatment groups: moxidectin (8 mg)/albendazole (400 mg) combination, ivermectin (200 µg/kg)/albendazole (400 mg) combination, albendazole (400 mg) monotherapy, ivermectin (200 µg/kg) monotherapy and moxidectin (8 mg) monotherapy.
b) to determine the CRs of the drug regimens against *T. trichiura*.
c) to evaluate the safety and tolerability of the treatments.
d) to determine the CRs and ERRs of the treatment schemes in study participants infected with hookworm and/or *A. lumbricoides*.
e) to investigate potential extended effects of the treatment regimens on follow-up helminth prevalence (5–6 weeks and 3 months post-treatment).
f) to characterize population PK parameters, as well as potential drug-drug interactions of active study treatments following single and co-administration in *T. trichiura* infected adolescents. If an exposure-response is observed, a PK/PD analysis will further be performed.

This article is reported in line with the Standard Protocol Items: Recommendations for Interventional Trials (SPIRIT) guidelines ^19^.

## Protocol

### Trial design

**Figure 1.**
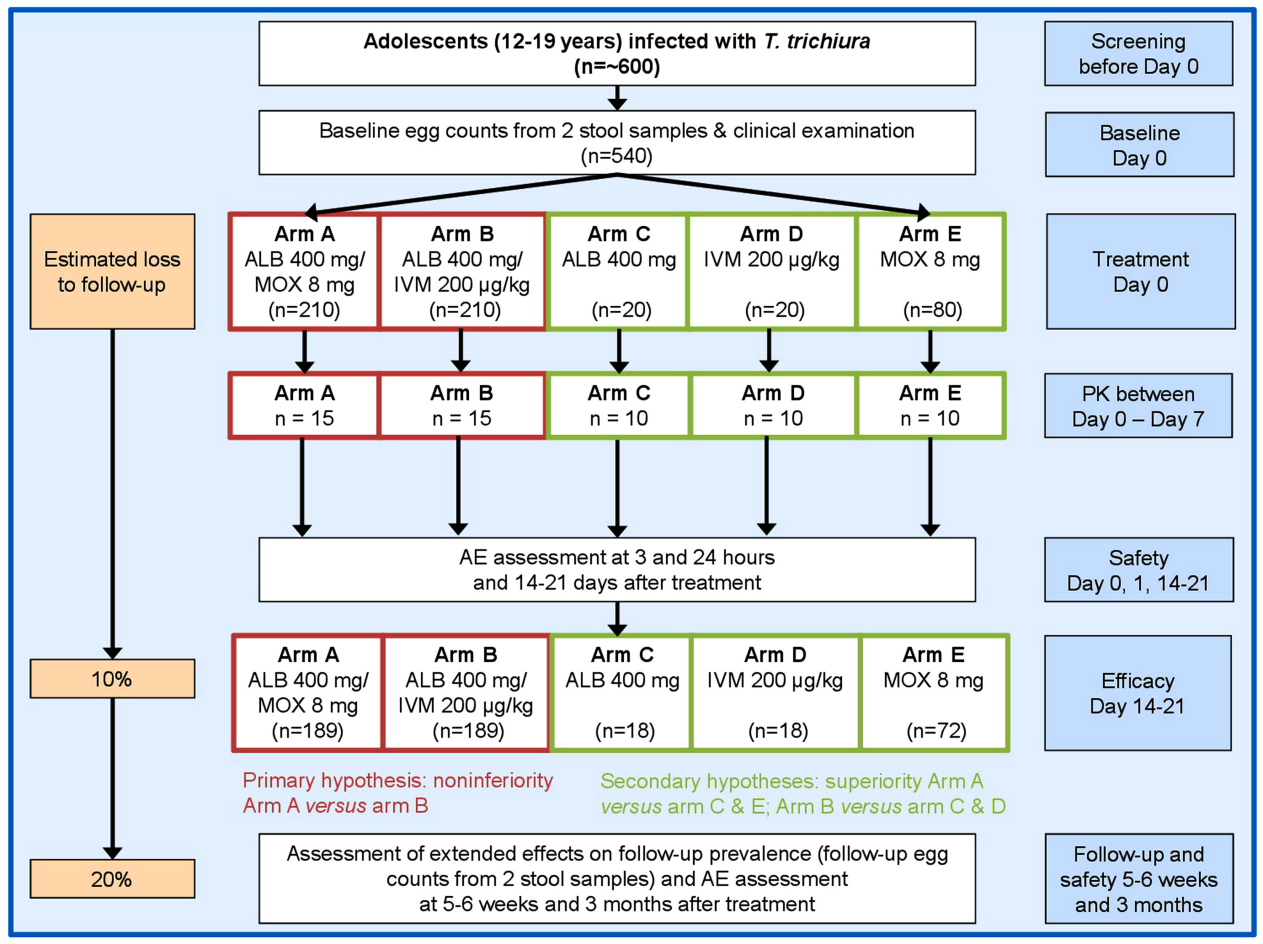
Design and timeline of the randomized controlled trial to be implemented on Pemba Island (Pemba Island, Tanzania). The study is designed as a five-arm trial including two arms with combined treatment through co-administration of separate tablets (arm **A**; moxidectin/albendazole, arm **B**; ivermectin/albendazole) and three arms with single drug administration (arm **C**; albendazole, arm **D**; ivermectin, arm **E**; moxidectin). Abbreviations: ALB, albendazole; MOX, moxidectin; IVM, ivermectin; PK, pharmacokinetics; AE, adverse events.

A phase 3 randomized controlled open-label non-inferiority trial with blinded outcome assessors will be conducted among adolescents aged 12–19 years with *T. trichiura* infection. The trial includes one baseline and three follow-up assessments at 14–21 days, 5–6 weeks and 3 months post-treatment ( Figure 1). The study is designed as a five-arm trial including two arms with combined treatment through co-administration of separate tablets (arm A; moxidectin/albendazole, arm B; ivermectin/albendazole) and three arms with single drug administration (arm C; albendazole, arm D; ivermectin, arm E; moxidectin). 

The efficacy of the treatments will be determined by collecting two stool samples before and at every post-treatment time-point. Each sample will be microscopically examined for *T. trichiura* eggs using duplicate Kato-Katz thick smears. Participants will be eligible if they are positive (≥ 48 eggs per gram of stool (EPG)) for *T. trichiura* eggs at baseline and will be considered cured at the different follow-up assessments if no *T. trichiura* eggs are found in the stool samples. 

### Outcomes

***Primary outcome.*** The primary outcome is the ERR of *T. trichiura* egg counts assessed by Kato-Katz microscopy pre-treatment and 14-21 days post-treatment.

***Secondary outcomes.*** The secondary outcomes include CR, defined as conversion from being egg positive pre-treatment to egg negative post-treatment, of *T. trichiura* as well as CRs and ERRs for *A. lumbricoides* and hookworm assessed by Kato-Katz at 14–21 days post-treatment. In addition, tolerability of treatment (type, number and severity of adverse events (AEs)), infection status and intensity derived by Kato-Katz at 5–6 weeks and 3 months post-treatment and PK/PD parameters of the study drugs will be assessed.

### Study area and participants

This trial will be implemented as a school-based study on Pemba Island (Zanzibar, on Pemba Island, Tanzania). Secondary schools in areas with moderate to high *T. trichiura* prevalence will be selected based on experience from earlier studies and/or knowledge of the local collaborating teams. These will be Kilindi, Kwale and Ndagoni located in Chake Chake district as well as Kiuyu in Wete district. In each selected school, adolescents aged 12–19 years will be invited for study participation. Entering school over-age is a common occurrence in Zanzibar, thus ages of secondary level pupils may well range from 12 to 19 years of age. Adolescents are within the main target group of helminth control programs and are listed among potential receivers of moxidectin that is, so far, only approved from 12 years of age onwards ^20^.

### Study duration

Screening for the baseline is scheduled to start 3 months prior to treatment. Follow-up screenings will take place between 14–21 days, 5–6 weeks and 3 months post-treatment and will last approximately two weeks, respectively. Thus, the maximum time for subject participation will be 6 months. The schedule of visits are summarized in Table 1.

**Table 1.** Schedule of visits during the study.

	Screening	Baseline/Treatment/Safety					Follow-up		
	Before day 0	0h		3h	24h	6h– 45h	14–21 days	5–6 weeks	3 months
**Informed consent**	X		**Randomization** **and treatment**						
**Diagnosis (stool** **examination)**	X						X	X	X
**Medical history**		X							
**Clinical examination**		X							
**Pregnancy testing**		X							X
**Hemoglobin ** **measurement**		X							
**PK (microsampling)**		X				X			
**Capturing AEs**				X	X		X	X	X
**Capturing SAE**				X	X		X	X	X

Abbreviations: PK, pharmacokinetics; AE, adverse events; SAE, serious adverse events

### Recruitment

School teachers and caregivers of potential participants and adolescents aged ≥18 years will be invited to participate in an information session. The research team will explain the purpose and procedures of the study, as well as potential benefits and risks of participation. Attendees will be encouraged to ask questions which will be discussed in an open setting. Caregivers interested in having their child/children of 12–17 years of age participate in the study or adolescents aged 18–19 years willing to participate will be invited to complete the process of informed consent by signing the informed consent form (ICF) ^19^. In addition, written assent will be obtained from underage participants. Participants having a signed ICF will be assessed for eligibility.

### Laboratory procedures

At baseline, all participants will be asked to provide two stool samples (within a maximum of seven days). From each stool specimen, duplicate Kato-Katz thick smears (41.7 mg each) ^21^ will be prepared and examined under a microscope for eggs of *T. trichiura*, *A. lumbricoides* and hookworm by skilled technicians.

For quality control of *T. trichiura* and *A. lumbricoides* egg counts, 10% of slides will be re-read by another laboratory technician. To ensure quality of hookworm diagnosis, 10% of the stool samples will be divided into two sub-samples; one of the containers will keep its original participant ID, whereas the second container will be labeled with a new ID (assigned by the co-PI). An additional Kato-Katz slide will be made from the second container and the findings compared to the ones from the original sample. For hookworm, results are considered correct if no difference in presence/absence of helminth eggs is found. For *T. trichiura* and *A. lumbricoides* the following tolerance margin should not be exceeded: (i) no difference in presence/absence of *T. trichiura* and *A. lumbricoides* (ii) egg counts are +/-10 eggs for counts ≤100 eggs or +/-20% for counts >100 eggs (for each species separately). In case discrepancies above the tolerance margin are noted, the respective slides are examined a third time. The new results are discussed to reach consensus. The same sampling procedure, diagnostic approach and quality control assessment will be applied at 14–21 days, 5–6 weeks and 3 months post-treatment.

### Eligibility criteria

Participants meeting all inclusion criteria and none of the exclusion criteria ( Table 2) will be invited for treatment.

**Table 2.** Inclusion and exclusion criteria.

Inclusion criteria	Exclusion criteria
Aged between 12 and 19 years. Written informed consent signed by either parents/caregivers for underage adolescents (aged 12–17 years) or by the participant him/herself (18–19 years of age); and written assent by underage participant. Agree to comply with study procedures, including provision of two stool samples at the beginning (baseline) and on three follow-up assessments (14–21 days, 5–6 weeks and 3 months after treatment). Willing to be examined by a study physician prior to treatment At least two slides of the quadruple Kato-Katz thick smears positive for *T. trichiura* and infection intensities of at least 48 EPG.	No written informed consent by individual or caregiver and/or no written assent by minors. Presence or signs of major systemic illnesses, e.g. body temperature ≥ 38°C, severe anemia (below 80g/l Hb according to WHO ^22^) upon initial clinical assessment. History of acute or severe chronic disease. Recent use of anthelmintic drug (within past 4 weeks). Attending other clinical trials during the study. Pregnancy, lactating and/or planning to become pregnant within the study period. Known allergy to study medications (i.e., albendazole, ivermectin or moxidectin).

Abbrevitations: EPG, eggs per gram of stool; Hb, hemoglobin; WHO, World Health Organization

### Clinical assessment

A clinical examination of the study participants assessing general health, anthropometric parameters including height and weight as well as forehead temperature using a Braun No touch + forehead NTF3000 (Braun GmbH, Kronberg, Germany) thermometer will precede the treatment. Each participant will be asked to provide a finger-prick blood sample for hemoglobin (Hb) levels, which will be measured using a HemoCue analyzer (Hb 301 system, Angelholm, Sweden). To avoid accidental treatment of pregnant girls/women all female participants will be asked to provide a urine sample for a pregnancy rapid diagnostic test at baseline and at the end of the study (3 months after treatment). Girls/women will be individually counselled that they should not become pregnant during the entire study period. All trial participants will further be asked about chronic diseases and existing clinical symptoms the day of exam, which will be evaluated on relevance with regard to exclusion criteria ( Table 2).

### Criteria for discontinuation of trial

A subject can be discontinued from the study for the following reasons:

a) The subject withdraws from the study: participation is fully voluntary; therefore withdrawal may happen anytime without further obligations.
b) At the discretion of the Principal Investigator (PI) or co-PI, if the participant is not compliant to the requirements of the protocol.

Discontinued subjects will not be replaced. If, for any reason, a subject is discontinued from the study after treatment but before the end of treatment evaluations, the safety assessment will still be conducted to ensure the discontinued participant’s well-being. Data obtained prior to the withdrawal will be included in the analysis to ensure the validity of the trial.

### Randomization, concealment and masking

Study participants eligible for treatment will be randomly assigned to one of the five treatment arms using a computer-generated stratified randomization code. The random allocation sequence will be generated by using an algorithm which minimizes deviations from the anticipated arm sizes stratified by 2 levels of baseline infection intensity (light: 1-999 EPG, and moderate/heavy plus heavy: ≥ 1000 EPG *T. trichiura* infections), which will be provided by the trial statistician not involved in enrolment, treatment and data collection. This ensures that all treatment arms will have a similar proportion of participants with light infection intensity. The number of light versus moderate/heavy infections, however, are not expected to be equal in each arm, depending on the distribution of infection intensity in the recruited cohort. Team members conducting the treatment will not know the allocation order. Concealment will be warranted by masking the randomization sequence using envelopes containing the respective treatment arm labels. The study is defined as open-label yet masking is assured since the primary outcome assessors, i.e., the microscopists determining the egg counts for the efficacy assessment, will have no knowledge of the participants’ assignment to treatment arms.

### Treatment

All eligible *T. trichiura*-infected participants will be treated with the respective single or combination treatment regimen at day zero. All regimens are administered orally as a single dose. 400 mg albendazole will be the product of Glaxo Smith Kline (Zentel®) and a single tablet administered. 3 mg tablets of ivermectin will be obtained from Merck (Stromectol®). The weight will be recorded for each participant and the correct dose (i.e., 200 µg/kg) evaluated and administered accordingly. Moxidectin 2 mg tablets will be obtained from Medicines Development for Global Health and 4 tablets administered to each participant. All drugs will be given in the presence of the investigator(s), and ingestion confirmed. This will be recorded with the time and date of administration. Subjects will be asked not to take any drugs other than those prescribed by the study medical team. After ingestion of the medication, the subjects will be observed for 3 hours to ensure retention of the drug. Vomiting and spitting within 1-hour post-dosing will require re-dosing. The subjects will not be allowed more than one repeated dose. No re-administration will be needed for subjects vomiting after one hour. The PI and/or Co-PI is responsible for drug accountability at the study site. Maintaining drug accountability includes careful and systematic study drug storage, handling, dispensing and documentation of administration.

At the end of the study all participants remaining positive for any STH infection will be treated with the currently best recommended treatment (i.e., ivermectin/albendazole against *T. trichiura* and hookworm and albendazole against *A. lumbricoides*).

### Pharmacokinetic studies

The PK study will be performed in a maximum of 15 participants in the combination chemotherapy treatment arms (i.e., arms A and B) and 10 participants in the monotherapy treatment arms (i.e., arms C-E), amounting to a subsample of 60 participants overall. All study participants, regardless of participation in PK, will receive a local high-fat breakfast before treatment ^17^. Since population PK parameters of all three study drugs are available ^23– 25^, a sparse sampling approach will be applied to describe the population-based PK profiles of the individual drugs upon mono- or co-administration. Additionally, potential interference between moxidectin or ivermectin and albendazole will be assessed. For this, capillary blood (≤60 µL) will be collected by puncture with a finger prick at four time points (approx. 6h, 21h, 27h, 45h post treatment). Two microsamples (duplicates) will be taken at each time point. Each time, the drop of blood will be directly transferred onto Mitra® sticks (Neoteryx, Toronto CA) (10 µL or 30 µL) and/or on Whatman® protein saver cards 903 filter paper (Merck, Darmstadt DE) (30 µL). Mitra® sticks will be utilized for participants having received albendazole and/or moxidectin, whereas filter paper will be used for ivermectin-treated participants. The dried Mitra® sticks and filter paper will be transported to Swiss TPH, Basel, and stored at room temperature until analysis within one month after blood collection. The quantification of the study drugs will be performed using validated liquid chromatography tandem mass spectrometry (LC-MS/MS) methods as described elsewhere ^23– 25^. Drug concentrations will be calculated by interpolation from a calibration curve with a lower limit of quantification of 1–5 ng/ml. 7% of the sample duplicates will be analyzed for quality control, and the measured concentrations will be used to determine between-run and overall precision and accuracy of the analysis.

### Safety assessments

Few adverse events (AEs) have been reported following albendazole, ivermectin or moxidectin single and co-administration in STH-infected individuals. The most common AEs were abdominal cramps, headache, itching, fatigue, nausea, diarrhea, fever and vertigo ^7– 9, 16, 26– 28^.

Interviews will be conducted to determine the emergence of clinical symptoms directly before treatment within the scope of baseline assessment. Participants will be kept for 3 hours after treatment administration to observe any possible acute AEs and reassessment will be done at 24 hours post-treatment. The local study physician will perform a full clinical examination if moderate to severe and/or unexpected AEs occur, and findings will be recorded. An emergency kit will be available on site to treat any medical conditions that warrant urgent medical intervention. At 3 and 24 hours after treatment and retrospectively at days 14–21 as well as 5–6 weeks and 3 months post-treatment, participants will again be interviewed for the assessment of AEs. Information on all AEs (incidence, intensity, seriousness and causality) will be entered immediately in the appropriate AE module of the case report form (CRF). For all AEs, sufficient information will be pursued and/or obtained so as to permit i) an adequate determination of the outcome of the event (i.e., whether the event should be classified as a serious adverse event (SAE)); and; ii) an assessment of the causal relationship between the AE and the study treatments. Intensity of AE will be judged by the study physician (active assessment) or a trained team member (retrospective assessment), following guidelines by the European Medicine Agency (ICH E2A Clinical safety data management: definitions and standards for expedited reporting) ^29^. Serious adverse events that are still ongoing at the end of the study period will be followed up to determine the final outcome. Any study-related unanticipated problem posing risk of harm to subjects or others (including all unexpected adverse drug reactions), and any type of SAE will be immediately (within a maximum of 24 hours after becoming aware of the event) notified to the study Sponsor-Investigator and co-PIs. Symptoms arising within the time span of 24 hours after treatment and the respective follow-up time points will be monitored passively by teachers or local health workers who will report incidences to the study team. All pregnancies will be reported to the Sponsor-Investigator promptly after becoming aware of the pregnancy. A study physician recruited from a local health facility/hospital will serve as medical contact between the study team and the treating physician or take up the role of treating physician directly. The treating physician will follow-up on the study participant until the end of the pregnancy (either by birth or resolved otherwise). The outcome of the pregnancy will be reported to the Sponsor-Investigator.

### Data management and data quality control

Investigators of Swiss TPH and Public Health Laboratory - Ivo de Carneri (PHL-IdC) have agreed on the protocol, performance of study procedures (SOPs from previous studies available on site), CRF completion, specimen collection and diagnostic methods prior to the initiation of the study.

CRF data will be double-entered and compared using Beyond Compare 4 (Scooter Software Inc., Madison, Wisconsin). Any discrepancies will be reviewed against the hard copies of the CRF and corrected accordingly. Electronic data files will be stored on secured network drives with restricted access to study personnel only. Data analysis will be conducted with pseudonymized data and reporting of findings will be fully anonymized.

***Source data.*** Source data are comprised of clinical findings and observations as well as laboratory data maintained and compiled at the study site. Source data are contained in source documents and are allowed to be accessed by local authorities. Source data will be directly entered in the following documents:

1. CRF: Primary data collection instrument for the study. It holds records of all clinical and physical examination data, treatment information and AEs. For every subject enrolled in the clinical trial, a corresponding CRF exists. All data requested on the CRF must be recorded, and investigators will review and approve each CRF for completion.
2. Laboratory parasitology sheets: Record of the STH egg counts at all sample collection time points
3. PK: Time records of PK samplings for 60 willing participants.

***Data collection and documentation.*** Data collected and produced within this trial will fall into one of the following categories:

a) Egg counts of *T. trichiura*, *A. lumbricoides* and hookworm ( *Necator americanus* and *Ancylostoma duodenale*, no differentiation between the two species will be made) derived from standard Kato-Katz microscopy performed at baseline as well as at 14–21 day, 5–6 weeks and 3 months post-treatment.
b) Anthropometric and clinical characteristics of the trial participants collected using the study’s CRF such as weight, height, blood pressure, temperature, pregnancy status (for female subjects), overall health status including any abnormal medical condition or chronic disease and AEs.
c) PK time recording of each sample per person.
d) Measured concentrations analyzed from micro blood samples and subsequently derived PK/PD parameters.

Data for categories a) to c) will be recorded both paper-based and directly into tablets using CommCare (Dimagi, Inc., Cambridge, MA) or computers, whereas data in category d) will be captured by software only. Data compiled using the software will be directly saved on the personal, password-protected laptop of one of the Co-PIs and uploaded to a server hosted at Swiss TPH, Basel. In paper-based data collection, all missing data must be explained. If an item on the CRF is left blank because the procedure was not done or the question was not asked “N/D” will be entered. If the item is not applicable to the individual case “N/A” will be written. All entries will be printed in black ink. All corrections must be noted with the initials of the respective team member and dated. Data in categories a) and b) will be merged into a masterfile and saved in .xlsx, .mdb and/or .csv. Paper-based data will serve as a physical backup and the source data. Data in categories c) and d) will be saved as .mdb, .csv, .xlsx, .txt and/or .pdf files.

***Data storage and preservation.*** All samples will be destroyed after completion of the study. Paper-based and/or electronic source data and related material will be preserved for a minimum of 15 years to enable understanding of the study procedures, which allows the work to be assessed retrospectively and repeated if necessary. The study site will retain a copy of the documents to ensure that local collaborators can provide access to the source documents to a monitor, auditor, or regulatory agency. Electronic source documents will be stored on a flash drive and kept at the study site (IdC PHL, Pemba Island, Tanzania). The primary data storage and backup will be in the Swiss TPH shared server and secondary data storage will be on personal, password-protected laptops. Electronic data files and archiving conditions will be made strictly confidential by password protection.

***Ethical, legal and confidentiality issues.*** Information about study subjects will be kept confidential and managed accordingly. Screened participants will be listed in a confidential “subject screening log” and attributed a unique study ID. In case of enrolment, participants will be listed in a confidential “subject enrolment log”; this document will constitute the only source to decode the pseudonymized data and will only be accessible to the investigators. Personal data will be coded for data analysis. No names will be published at any time, and published reports will not allow for identification of single subjects. Confidentiality will be ensured throughout the entire research project. All databases will be password secured. None of the investigators declare to have any conflicts of interest.

### Statistics

***Sample size calculation.*** For the primary analysis the trial is designed as two arm parallel group randomized controlled trial. We test the primary hypothesis that the treatment combination moxidectin and albendazole is not inferior compared to ivermectin and albendazole. To determine the required sample size, we run a series of simulations using artificial data which behaved roughly in the same way as found by Barda *et al.* ^8^. Assuming true ERR of 98% in both arms, we estimate that 160 participants are required in each group to be at least 90% sure that the limits of a two-sided 95% confidence interval (CI) will exclude a difference in favor of the standard group of more than 2 percentage points. To account for a potential loss to follow-up of 10% and including a safety margin of 20% to account for uncertainty in our assumptions underlying the simulations, we anticipate enrolling 210 participants in each combination treatment arm (arm A and B). The secondary hypothesis anticipates superiority of combination therapies against monotherapies. Assuming CRs below 25% for albendazole as well as for ivermectin and 40% for moxidectin monotherapy, we need to enroll 20, 20 and 80 adolescents, respectively, to identify a statistical significant difference with 85% to 90% power (arm C, D, E) ^1, 6, 9, 30– 32^. We thus aim to recruit 210 + 210 + 20 + 20 + 80 = 540 participants in total.

The suggested sample size of a maximum of 4 PK time points from 60 willing participants (10–15 per study arm) is sufficiently high to determine the population PK parameters and investigate potential drug-drug interactions with a sparse sampling scheme, considering that PK variability is moderate. A moderate PK variability is a reasonable assumption when dealing with adolescents.

***Description of statistical methods.*** In non-inferiority trials, non-inferiority has to be demonstrated in the intention-to-treat and in the per protocol population. The primary analysis will be performed according to the intention-to-treat principles using the available case population, which includes all participants with any primary end point data. Subsequently, a per-protocol analysis will be performed. Eggs per gram of stool will be assessed by calculating the mean egg count from the quadruplicate Kato-Katz thick smears and multiplying this number by a factor of 24. The geometric mean (GM) ERR will be calculated as:

$$ERR_{GM}=1-\frac{e^{\frac{1}{n}\sum \log(EPG_{follow-up}+1)}-1}{e^{\frac{1}{n}\sum \log(EPG_{baseline}+1)}-1}$$

GM egg counts will be calculated for the different treatment arms before and at 14–21 days after treatment to assess the corresponding ERRs. Bootstrap resampling method with 5,000 replicates will be used to calculate 95% CIs for ERRs and the difference between the ERRs.

CRs will be calculated as the percentage of egg-positive adolescents at baseline who become egg-negative after treatment. Differences among CRs will be assessed by using unadjusted logistic regressions. In a subsequent analysis an adjusted logistic regression (adjustment for baseline infection intensity, age, sex, weight) will be performed. ERRs and CRs as well as their respective confidence intervals will be calculated separately for each follow-up. Statistical analysis will be done using R version 4.0.3 (R Foundation, Vienna, Austria).

AEs will be summarized descriptively in tables and figures providing information on clinical relevance, timing, frequency, type, severity and causality by treatment arm.

A nonlinear mixed-effects (NLME) modelling will be used to determine PK parameters including absorption rate (k _a_), volume of distribution (V), and clearance (CL). Concentrations are measured with a validated LC-MS/MS method ^23– 25^. Using non linear mixed effects, the key population PK parameters will be calculated based on which an effect on the drug-drug interaction might be determined:

- C _max_ maximal plasma concentration
- t _max_ time to reach C _max_
- AUC area under the curve, from 0 to last time point and 0 to inf.
- t _1/2_ elimination half-life

C _max_ and t _max_ will be observed values derived from the plasma concentration-time profile. Total drug exposure (AUC) and t _1/2_ will be calculated with the NLME modeling software Monolix 2018R2 (Lixoft, Antony, France) using compartmental analysis. The elimination half-life will be estimated by the equation: t _1/2_ = ln2/λ, where λ (the elimination rate constant) will be determined by performing a regression of the natural logarithm of the concentration values during the elimination period.

### Ethical considerations

***Independent ethics committee.*** The study has been reviewed and approved by the institutional research commission of the Swiss TPH, the ethics committee in Switzerland: ‘Ethikkomission Nordwest- und Zentralschweiz’ (AO_2020-00042; date of approval 24 November 2020), and the ‘Zanzibar Health Research Ethics Review Committee’ (reference no. ZAHREC/03/PR/OCT/2020/23; date of approval 22 October 2020). The study will be undertaken in accordance with the Declaration of Helsinki and good clinical practice. Material transfer agreements between the PHL-IdC and Swiss TPH will regulate the transfer of collected samples.

***Evaluation of the risk-benefit ratio.*** Albendazole, ivermectin and moxidectin are well-known drugs and have little and mainly mild AEs as described to date (e.g., headache, abdominal pain) ^6, 9, 16, 26– 28, 33^. Albendazole and ivermectin are widely used drugs in mass treatment programs against filariasis while only moxidectin is a relatively new drug, FDA-approved against onchocerciasis ^34^. All community members enrolled in the study will benefit from a clinical examination and a treatment against STHs. All participating subjects remaining positive for *T. trichiura* will be treated with ivermectin (200 μg/kg)/albendazole (400 mg), considering this combination showed higher efficacy compared to the existing standard treatment (albendazole alone) and the recent inclusion of ivermectin-albendazole as recommended treatment scheme against STH on the List of Essential Medicines ^4^.

***Subject information and consent.*** Information sessions at the respective schools will be conducted to explain to teachers, caregivers and potential participants the purpose and procedures of the study, as well as potential benefits and risks of participation. All parents or caregivers of eligible adolescents and all participants ≥18 years will be invited to sign a written informed consent sheet. In case the person is illiterate, an impartial witness that can read and write has to sign the consent and the illiterate participant has to give a thumb print. Parents or caregivers and adult participants will have sufficient time for reflection of their child’s or their own participation, respectively. Additionally, adolescents (aged 12–17 years) will be briefed verbally, and written assent will be sought in form of their name and signature written down or if illiterate by providing a thumbprint.

Parents or caregivers attending this meeting will receive a small provision to cover their costs for transportation (~US$ 2). Participation is voluntary and individuals have the right to withdraw from the study at any given point in time with no further obligations. Participation itself will not be awarded with compensation.

### Quality control and quality assurance

We will work with a locally based external monitor, who will conduct site visits to the investigational facilities for the purpose of monitoring the study. Details will be described in a separate monitoring plan. The investigator will permit them access to study documentation and the clinical supplies dispensing and storage area. Monitoring observations and findings will be documented and communicated to appropriate study personnel and management. A corrective and preventative action plan will be requested and documented in response to any significant deviation. No sponsor-initiated audits are foreseen, but audits and inspections may be conducted by the local regulatory authorities or ethics committees. The investigator agrees to allow inspectors from regulatory agencies to review records and is encouraged to assist the inspectors in their duties, if requested.

In our study, no data and safety monitoring board will be established, since we work with well-known drugs in a small sample size and using a single dose treatment. However, advisors will be informed regularly and the findings discussed.

### Dissemination of study results and publication

The final results of this study will be published in a scientific journal and presented at scientific conferences. The Bill & Melinda Gates Foundation will be acknowledged as study funder. All results from this investigation are considered confidential and shall not be made available to any third party by any member of the investigating team before publication. A summary of study conclusions will be shared with ZAHREC. After publication, study results will be made available to study communities.

### Study status

The screening phase to identify eligible trial participants was initiated in March 2021.

## Discussion

Building on our previous work, which identified moxidectin/albendazole as a promising treatment for STH infections and determined the ideal doses ^8, 9^, a randomized controlled non-inferiority trial with five treatment arms will be carried out, testing the performance and safety of co-administered moxidectin and albendazole versus combined ivermectin and albendazole treatment. Both combinations will be compared to monotherapy of albendazole, moxidectin and ivermectin. To our knowledge, this trial marks the first randomized controlled trial assessing the safety and efficacy of combined moxidectin/albendazole compared to the recently recommended co-administration of ivermectin/albendazole against STH infections. The evidence from this trial on the efficacy, safety and potentially prolonged infection clearance in adolescents as part of the main target groups may provide further guidance to STH control programs.

Pharmacokinetic evaluations provide critical information about how the individual body responds to the drug, and is most often influenced by dietary habits, gender, age, body mass index, ethnicity, and/or infection type. Our objective is to characterize population PK parameters and drug-drug interactions of the active study treatments after single and co-administration in *T. trichiura*-infected adolescents in on Pemba Island. This study will describe the PK parameters for the first time of any of the study drugs in this population, of ivermectin and moxidectin in this age group and of moxidectin for this infection type overall. The results will inform whether the findings of this study can be directly applied to other treatment cohorts and will prevent drug failure for predictable reasons in future clinical trials. Drawing from the extensive experience from our research group, the protocol provides the clear outline for a randomized controlled trial that will provide high-quality data on the efficacy and safety of the two drug combinations as well as potential long-term effects. The comparatively long screening time between enrolment of the first participants and treatment may present a limitation, however we anticipate possible fluctuations in egg counts to occur evenly throughout the study group and within the anticipated limits controlled by the inclusion criterion of presenting with at least 48 EPG and positivity on at least two out of four Kato-Katz slides.

## Conclusion

This trial aims to inform decision- and policymakers on how and which anthelminthic combination therapy could be introduced into existing large-scale deworming campaigns and thus provide a valuable adjunct tool for interrupting STH transmission and delay potential selection of drug resistance.

## Data availability

### Underlying data

No data are associated with this article.

### Extended data

Open Science Framework: Efficacy and Safety of MOX/ALB vs. IVM/ALB co-administration. https://doi.org/10.17605/OSF.IO/A3N85 ^19^.

*This project contains the following extended data:*

- Additional file 1_WHO-trial-reg-dataset_Moxi-ALB_IVM-ALB_combi trial_2021-06-11_OSF.pdf (World Health Organization trial registration data set)
- Additional file 2_ICF_Moxi-ALB_IVM-ALB_combi trial_v1.0_2020-06-15_OSF.pdf (Participant information and consent sheet)

### Reporting guidelines

Open Science Framework: SPIRIT checklist for “Efficacy and safety of moxidectin and albendazole compared to ivermectin and albendazole co-administration in adolescents infected with *Trichuris trichiura*: a randomized controlled trial”. https://doi.org/10.17605/OSF.IO/A3N85 ^19^.

Data are available under the terms of the Creative Commons Zero "No rights reserved" data waiver (CC0 1.0 Public domain dedication).
