# Supplementary material for: Efficacy and safety of moxidectin and albendazole compared to ivermectin and albendazole co-administration in adolescents infected with Trichuris trichiura: a randomized controlled trial protocol
Source: Gates Open Res. 2021 Sep 27;5:106. Originally published 2021 Jul 21. [Version 2] doi: 10.12688/gatesopenres.13299.2 (PMC8488464; doi:10.12688/gatesopenres.13299.2)
Supplement: Peer review history

# Peer review: Reviewer response for version 2

**DOI:** 10.21956/gatesopenres.14639.r31216

**Published:** 2021-10-01

**Authors:** Judd L. Walson

**Affiliations:** 1 Department of Global Health, University of Washington, Seattle, WA, USA

The authors have responded to the comments provided by this reviewer. I appreciate the careful attention to the responses and I feel as though the concerns raised have been reasonably addressed and the limitations acknowledged.

Is the study design appropriate for the research question?

Yes

Is the rationale for, and objectives of, the study clearly described?

Yes

Are sufficient details of the methods provided to allow replication by others?

Yes

Are the datasets clearly presented in a useable and accessible format?

Not applicable

Reviewer Expertise:

Infectious Disease, NTDs, STH, epidemiology, clinical research

I confirm that I have read this submission and believe that I have an appropriate level of expertise to confirm that it is of an acceptable scientific standard.

# Peer review: Reviewer response for version 2

**DOI:** 10.21956/gatesopenres.14639.r31217

**Published:** 2021-10-01

**Authors:** Alejandro Krolewiecki, Pedro Fleitas

**Affiliations:** 1 Instituto de Investigaciones de Enfermedades Tropicales (IIET-CONICET), Sede Regional Orán, Universidad Nacional de Salta, Orán, Argentina; 2 Instituto de Investigaciones de Enfermedades Tropicales (IIET-CONICET), Sede Regional Orán, Universidad Nacional de Salta, Orán, Argentina

All comments and questions have been adequately addressed by the authors. The manuscript is clear and is informative on the justification, aims and methodology.

Is the study design appropriate for the research question?

Yes

Is the rationale for, and objectives of, the study clearly described?

Yes

Are sufficient details of the methods provided to allow replication by others?

Yes

Are the datasets clearly presented in a useable and accessible format?

Not applicable

Reviewer Expertise:

Infectious diseases, Neglected Tropical Diseases, Clinical Research

We confirm that we have read this submission and believe that we have an appropriate level of expertise to confirm that it is of an acceptable scientific standard.

# Peer review: Reviewer response for version 1

**DOI:** 10.21956/gatesopenres.14540.r31098

**Published:** 2021-09-06

**Authors:** Alejandro Krolewiecki, Pedro Fleitas

**Affiliations:** 1 Instituto de Investigaciones de Enfermedades Tropicales (IIET-CONICET), Sede Regional Orán, Universidad Nacional de Salta, Orán, Argentina; 2 Instituto de Investigaciones de Enfermedades Tropicales (IIET-CONICET), Sede Regional Orán, Universidad Nacional de Salta, Orán, Argentina

The manuscript by Welsche et al describes the study protocol of a RCT for the treatment of *Trichuris trichiura* infections in adolescents with combination treatment of albendazol with either moxidectin or ivermectin, as well as each of these three drugs as monotherapy. It is a relevant question with implications for the public health control of STH in general and *T. trichiura* in particular. The justification of the trial, study design and selected population are well described and the primary objective with its corresponding outcome measurement are well described.

 Comments for the authors:

 Suggest including in the abstract and “Trial design” sub sections a mention to the blinded outcome assessor feature of the trial.

 The description of the diet to be administered with the treatments should be clarified. It would be useful to include information on the amount of total calories and/or grams of fat content of the “local high fat diet”. It is not clear whether that diet will accompany only the PK participants or the whole study population.

 The quality control plan for discordant samples is different for hookworm than for the other species; could explain the reason for that difference.

 Table 2. The exclusion criteria mentioning medication with known interaction with study drugs should include a list of those drugs.

 Secondary objectives referring to the comparison between diagnostic methods lacks a description of the methodologic approach to its statistical analysis.

 Suggest providing more information on the baseline prevalence reported in the different schools/districts. If there are significant differences between these prevalence, generalized linear mixed models with the school and/or district as random effects should be used, rather than logistic regression, to assess the CRs at the 5-6 weeks and 3 months time points. A description of the analyzes to be carried out at the different time points is also required.

 The inclusion of a group receiving ivermectin monotherapy does not appear as justified in order to test any of the relevant questions; but this comment is just a personal view.

 Clarify the time interval allowed between both stool samples at screening and at the 3 other time-points (unclear if in the post-treatment visits 1 or 2 samples will be collected).

 Suggest including references that endorse the assumed CRs for albendazole and ivermectin monotherapy (Sample size calculation subsection).

Is the study design appropriate for the research question?

Yes

Is the rationale for, and objectives of, the study clearly described?

Yes

Are sufficient details of the methods provided to allow replication by others?

Yes

Are the datasets clearly presented in a useable and accessible format?

Not applicable

Reviewer Expertise:

Infectious diseases, Neglected Tropical Diseases, Clinical Research

We confirm that we have read this submission and believe that we have an appropriate level of expertise to confirm that it is of an acceptable scientific standard, however we have significant reservations, as outlined above.

# Response

**Published:** 2021-09-10

**Authors:** Jennifer Keiser

**Affiliations:** Swiss Tropical and Public Health Institute, Switzerland

1. *Abstract and trial design -> mention blinded outcome assessor*

Will be included in version 2

1. *Description of diet*

The information on calories and grams of fat content is unfortunately not available to us as we will get the food fresh from a local cook. We will provide all participants, not just the ones taking part in PK, with two maandazi (comparable to doughnuts) and sugared tea prior to drug administration. This food was chosen after consulting the students on their preferences to make sure participants will really eat what we provided them. A clarification was added in version 2 (please see “Pharmacokinetic studies”).

1. *Different quality control plan for discordant results Hookworm vs. T. trichiura / Ascaris*

Hookworm eggs dissolve within a relatively short time after slide preparation, whereas T. trichiura and A. lumbricoides eggs stay visible. Therefore, hookworm QC has to be done directly after slides are prepared. For logistical and practicality reasons, a separate slide is prepared for hookworm reading instead of disturbing the sample flow by picking out slides. For T. trichiura and A. lumbricoides however, it is possible to select slides for QC readings after all samples have been processed to have them read the next day. The quality control margins are different for hookworm QC, i.e. only positive or negative, to account for the variance between the original slides and the new slides prepared.

1. *Medication with interactions in exclusion criteria*

This part will be taken out in version 2, it was an artefact from a previous version. Also please see answer to comment 12 of review 1.

1. *Diagnostic methods*

The lack of exact descriptions was due to the experimental nature of the application of FECPAK G2, which was intended to be done as a proof of concept in close collaboration with TECHION, whose technicians are constantly developing the platform. Unfortunately, the Micro-I devices faced technical issues which will make it impossible for us to include the device in the study. See also comment 1 of review 1.

1. *Baseline prevalence between schools and description of analysis as FU timepoints*

We will follow the first suggestion of the reviewer and will provide the main baseline characteristics (age, sex, baseline infection intensity) stratified by school.

 With respect to a random effect model the reviewer raised an interesting point. Random effect models are especially recommended in multi-center trials if the number of clusters is high and/or the baseline risk is likely to vary substantially across centers and/or centers are likely to influence patient outcomes. The number of centers in our case is only 4 and neither the baseline risk (geographical proximity) or treatment outcome (same study personnel) is expected to vary noteworthy among centers. However, there is another important point: egg reduction rates cannot be approximated by any theoretical distribution (although zero inflated negative binomial models have been suggested, we do not think that this appropriate, e.g. because of treatment failures). We construct the confidence intervals using bootstrap re-sampling approach which is difficult to combine with random effects.

 The reviewer is right that we did not specify which analysis is done at each time point. This is clarified in the revised version.

1. *Ivermectin monotherapy arm*

We had several internal discussions if all trial arms are required but we finally decided to follow the standard guidelines for combination therapies: 'each substance of a fixed combination must have documented contribution within the combination'

1. *Time interval allowed between both stool samples*

At all time-points, two samples will be collected which should be provided not more than 7 days apart (please see “Laboratory procedures”)

1. *References endorsing the assumed CRs for ALB and Ivm monotherapy*

Will be included in version 2.

 Belizario et al., 2003, A comparison of the efficacy of single doses of albendazole, ivermectin and diethylcarbamazine alone or in combinations against Ascaris and Trichuris spp.

 Marti et al., 1996, A comparative trial of single-dose ivermectin versus three days of albendazole for treatment of Strongyloides stercoralis and other soil-transmitted helminth infections in children

# Peer review: Reviewer response for version 1

**DOI:** 10.21956/gatesopenres.14540.r30940

**Published:** 2021-07-27

**Authors:** Judd L. Walson

**Affiliations:** 1 Department of Global Health, University of Washington, Seattle, WA, USA

Updated on 01/10/21 to include a competing interest statement

 This paper presents the protocol for a randomized trial of moxidectin/albendazole vs. ivermectin/albendazole for the treatment of adolescents with Trichuris. The study is timely and will be an important contribution to the literature. Overall, the study is well designed, although there are some minor issues that should be considered.

 One major concern is the attempt to include a nested study of alternative diagnostics in the trial. The primary endpoint of the trial is dependent on KK and the inclusion of qPCR and FECPAK are really separate exploratory studies that should probably be removed from the primary protocol. I think that these only serve to add confusion to the protocol as it is not entirely clear how discrepancies in results using these various methods would be resolved. I would suggest removing mention of these alternative diagnostics and presenting that as a separate sub-study.

 More justification regarding why an alternative 2 drug regimen is needed should be provided. Moxi/alb would likely be significantly more expensive, require a new supply chain, not be part of the existing drug donation program, etc. Given these issues, is non-inferiority even relevant? One might argue that you need to demonstrate superiority to overcome these issues. I think having data on an alternative regimen is worthwhile but these limitations should be discussed.

 The introduction should remove the paragraph about the BMGF convening and the desires of the funding sponsored group. This is not really relevant to the research and the authors should explain the rationale from a scientific need – not a funder driven priority standpoint.

 The authors suggest that this would be a phase III study. I think this is more a phase IIb study and would not, on its own, change policy. Consider reframing this as a phase IIb.

 The authors should be more clear that this is a single blind study (lab staff are blind to treatment allocation). It is not clear why this was not designed as a double-blind study – this would significantly strengthen the design and I see no rationale for not doing that?

 The authors state that participants are eligible if they are positive for trichuris – is this in either of the two samples or in both?

 More detail as to how AEs are being measured is needed. Is this passive – it seems to be somewhat active through the CRFs but more detail is needed.

 Having screening 3 months before baseline is problematic and it is not clear why this would be done this way. In addition, at low counts, one would imagine that a sizable fraction of those who screen positive will subsequently be negative at baseline – is this reflected in planning to enroll the necessary sample size?

 It is likely that there will be a larger number of participants at the lower range of the age – how is this accounted for in the analysis?

 It appears as though discordant sample results are resolved through consensus – this is not terribly scientific. Would encourage clear SOP based rules for resolving discrepancies.

 It appears that all girls/women will undergo a pregnancy test and hemoglobins will also be measured. Please provide information about how the results of these tests will be handled? Will parents be told of pregnancy results? There are serious ethical issues to consider here – please expand.

 How will concurrent medication use be assessed and which are exclusion criteria?

 Given the impact of diet on PK of these drugs, will dietary history be collected?

 Please confirm that a DSMC will be assembled and confirm whether an interim analysis will be conducted and if there are any stopping rules for harm?

 The fact that the protocol has not been agreed by all investigators (under data management and quality control) is concerning and suggests that the collaborators have not participated fully in the development of this protocol. Please clarify.

 Please provide more detail re QA/QC plans for the lab work and the anthropometry.

 Please confirm that the trial has been registered on clinicaltrials.gov or similar.

 It appears that only individuals with endpoint data will be included – this is a modified ITT. Everyone randomized should be included in the analysis.

Is the study design appropriate for the research question?

Yes

Is the rationale for, and objectives of, the study clearly described?

Yes

Are sufficient details of the methods provided to allow replication by others?

Yes

Are the datasets clearly presented in a useable and accessible format?

Not applicable

Reviewer Expertise:

Infectious Disease, NTDs, STH, epidemiology, clinical research

I confirm that I have read this submission and believe that I have an appropriate level of expertise to confirm that it is of an acceptable scientific standard, however I have significant reservations, as outlined above.

# Response

**Published:** 2021-09-10

**Authors:** Jennifer Keiser

**Affiliations:** Swiss Tropical and Public Health Institute, Switzerland

1. *nested study of alternative diagnostics*

We agree with the reviewer’s suggestion to remove mention of the alternative diagnostics from this publication. For reference of the original protocol, which received approval from the ethical committees, please consult the registration at clinicaltrials.gov

1. *justification regarding why an alternative 2 drug regimen is needed*

Non-inferiority is warranted since the goal of the study is not to show that mox/alb is a superior treatment but rather to demonstrate moxidectin to be a valid alternative to ivermectin to broaden the pool of possible treatment options in light of ivermectin being already broadly administered for other parasitic diseases with potential resistance-development in the near future. Mox/Alb might also be useful treating *T. trichiura* infections not responding to iver/alb.  Of note, ivermectin is also not a cheap drug and moxidectin will likely be widely used in the future.

1. *remove the paragraph about the BMGF convening and the desires of the funding sponsored group from introduction*

We agree with the reviewer and removed these sections from version 2.

1. *Phase IIb vs. phase III study*

Arguably the clinical trial has elements of both phase IIb (limited number of participants and safety regards) and phase III trials (randomization, comparison to standard drug, drug already approved for onchocerciasis, efficacy). The principal and all co-investigator decided on designating it as phase III study in the protocol, which was approved by the ethical committees.

1. *Single blind*

The study is open-label because both the study subjects and the administering investigator know which treatment is given, however unbiased results are assured by the laboratory technicians being blinded to the allocation. The participants are not explicitly informed of the treatment and they will not know which of the two combination treatments they receive (arms A and B), but by the number of tablets and their size they may be able to detect if they are in a mono-arm or combination arm. Double-blinding or even full single-blinding would have meant having to administer a large amount of placebo pills: an albendazole-placebo as well as moxidectin/ivermectin placebos. We discussed blinding at length internally and deemed it more ethical and practical to reduce the number of pills administered rather than having full blinding.

1. *Trichuris positive:*

Inclusion criteria: a mean epg of at least 48 over both samples and positive in at least two out of 4 Kato-Katz slides

1. *More detail as to how AEs are being measured*

Actively: at 3h and 24h, performed by the study physicians. Passively/retrospectively: at 14-21 days, 5-6weeks and 3months post-treatment, performed by trained study personnel and in case of occurring AEs the study physicians are consulted.

1. *Screening 3 months before baseline*

The screening period was set as three months in the protocol given that this trial will be performed under difficult conditions during a pandemic. Obviously we will try to conduct the baseline screening as quickly as possible. For practicality reasons, treatment will only be done once screening for all potential participants is concluded. Given the historically consistent high prevalence of *T. trichiura* infection in the study population and the minimum requirement of 48epg over two samples, we do not expect spontaneous clearance of infection by beginning of treatment. A safety margin of 20% was included in the sample size calculations.

1. *Larger number of participants at the lower range of age*

In fact, the largest number of participants are likely to be in the medium age range (14-16 years) due to recruitment in secondary schools and the overall high prevalence of *T. trichiura* infections across all age-groups. The analysis does not account for stratification by age or sex. Clinical trials are geared towards internal validity and do not aim at being representative for the whole population. Internal validity is warranted due to equal distribution of age and sex among treatment arms.

1. *Discordant results resolved through consensus*

Slides not passing QC are re-read and results accepted on the basis of all three readings. If the results are discordant after the third reading, the slide is read by the technician performing the QC reading with the microscope connected to a screen to enable all technicians to witness the reading and the result accepted after the final joint reading.

1. *Handling of pregnancy results*

The local Co-PI was consulted to establish the ethically best approach. Unexpected pregnancies are to be discussed privately among the study physicians and the participant. In case of the participant being minor (below 18 years), the caregiver/parent will be informed by the study physician with the participant’s wishes being taken into account as much as possible. The matter will be handled with utmost discretion and results not communicated to anyone but the participant and if minor the caregiver/parent. 

1. *Concurrent medication*

The study physicians will assess concomitant medication of each participant prior to treatment during clinical examination. Participants will be excluded if concomitant medication is taken for a severe acute or chronic disease or if anthelminthics have been taken during the last 4 weeks. There are no clinically relevant drug interactions known with any of the study drugs, rare reports of an increased International Normalized Ratio have been reported if ivermectin is co-administered with warfarin. In version 2, this section has been removed (Please see exclusion criteria).

1. *Dietary history*

Participants will be asked to arrive fasted on treatment day, where everyone will receive the same fatty breakfast. The absorption of the drug is directly related to the youngest dietary history, which is why every subject will be treated alike. Fat has been reported to increase the absorption by a factor of two for certain drugs.

 Dietary history of more than 12h does contribute to drug metabolism (e.g. malnutrition), as do generally all genetic and environmental factors. This is why it is important to assess PK in different populations.

1. *DSMC*

STH trials can be completed in a few weeks/months, in particular in a high prevalence site such as Pemba, with a treatment phase of just a few days. Furthermore, the study drugs are well known, a DSMB was therefore not assembled in this study.

1. *Protocol not agreed by all investigators*

We do not understand this comment. All investigators have agreed on the protocol prior to study initiation and all collaborators were fully involved in development.

1. *QA/QC details*

Please see sections ‘Laboratory procedures’ (p. 4-5) and ‘Data management and data quality control’ (p. 8-9) for detailed descriptions.

1. *Clinicaltrials.gov*

Please see abstract (NCT04700423)

1. *Modified ITT vs. per-protocol*

The reviewer raises an important point. Missing data might result in biased estimates. Several approaches have been proposed to reduce the potential impact of missing data including Expectation Maximization algorithms, Full Information Maximum Likelihood estimation, Multiple Imputation or the less sophisticated Mixed Models for Repeated Measures. Unfortunately, none of these can be used together with egg reduction rates. In addition, previous trials have shown that all observed covariates except baseline infection intensity showed no noteworthy association with the outcome or the the probability of being missing. Therefore, the usefulness of the aforementioned approaches remains questionable.   Consequently, we use the next best option the available case analysis. We do not use the term modified ITT because this term, has been used inconsistently and often refers to a per protocol analysis (Abraha et al. 2010 doi.org/10.1136/bmj.c2697). There are good reasons to believe that the available case analysis provides in our case valid estimates: i) the proportion of participants with missing data is expected to be low (around 5%), ii) given the nature of the disease and the usually mild adverse events associated with the drugs we can assume that most missing data follow a missing completely at random pattern, and iii) in a non-inferiority trial the ITT analysis is not considered the gold standard as in superiority trials but is one analysis which has to interpreted together with the estimates from the per protocol population.
